# A mixture of 13 pesticides, contaminants, and food additives below individual NOAELs produces histopathological and organ weight changes in rats

**DOI:** 10.1007/s00204-023-03455-x

**Published:** 2023-03-09

**Authors:** Viorica Dinca, Anca Oana Docea, Andrei Ioan Drocas, Taxiarchis Konstantinos Nikolouzakis, Polychronis D. Stivaktakis, Dragana Nikitovic, Kirill S. Golokhvast, Antonio F. Hernandez, Daniela Calina, Aristidis Tsatsakis

**Affiliations:** 1 Doctoral School, University of Medicine and Pharmacy of Craiova, 200349 Craiova, Romania; 2 Department of Toxicology, University of Medicine and Pharmacy of Craiova, 200349 Craiova, Romania; 3 Department of Urology, University of Medicine and Pharmacy of Craiova, 200349 Craiova, Romania; 4 Laboratory of Toxicology, Medical School, University of Crete, 71003 Heraklion, Greece; 5 Laboratory of Histology-Embryology, Medical School, University of Crete, 71303 Heraklion, Greece; 6 Siberian Federal Scientific Center for Agrobiotechnology RAS, Krasnoobsk, Russia; 7 Department of Legal Medicine and Toxicology, University of Granada School of Medicine, Granada, Spain; 8 Instituto de Investigación Biosanitaria Ibs. GRANADA, Granada, Spain; 9 Consortium for Biomedical Research in Epidemiology & Public Health (CIBERESP), Barcelona, Spain; 10 Department of Clinical Pharmacy, University of Medicine and Pharmacy of Craiova, Craiova, Romania

**Keywords:** Mixtures, Pesticides, Low-dose toxicity, Cytotoxicity, Food additives

## Abstract

The current approach for the risk assessment of chemicals does not account for the complex human real-life exposure scenarios. Exposure to chemical mixtures in everyday life has raised scientific, regulatory, and societal concerns in recent years. Several studies aiming to identify the safety limits of chemical mixtures determined hazardous levels lower than those of separate chemicals. Following these observations, this study built on the standards set by the real-life risk simulation (RLRS) scenario and investigated the effect of long-term exposure (18 months) to a mixture of 13 chemicals (methomyl, triadimefon, dimethoate, glyphosate, carbaryl, methyl parathion, aspartame, sodium benzoate, EDTA, ethylparaben, butylparaben, bisphenol A and acacia gum) in adult rats. Animals were divided into four dosing groups [0xNOAEL (control), 0.0025xNOAEL (low dose—LD), 0.01xNOAEL (medium dose—MD) and 0.05xNOAEL (high dose-HD) (mg/kg BW/day)]. After 18 months of exposure, all animals were sacrificed, and their organs were harvested, weighed, and pathologically examined. While organ weight tended to be higher in males than in females, when sex and dose were taken into account, lungs and hearts from female rats had significantly greater weight than that of males. This discrepancy was more obvious in the LD group. Histopathology showed that long-term exposure to the chemical mixture selected for this study caused dose-dependent changes in all examined organs. The main organs that contribute to chemical biotransformation and clearance (liver, kidneys, and lungs) consistently presented histopathological changes following exposure to the chemical mixture. In conclusion, exposure to very low doses (below the NOAEL) of the tested mixture for 18 months induced histopathological lesions and cytotoxic effects in a dose and tissue-dependent manner.

## Introduction

**Fig. 1:**
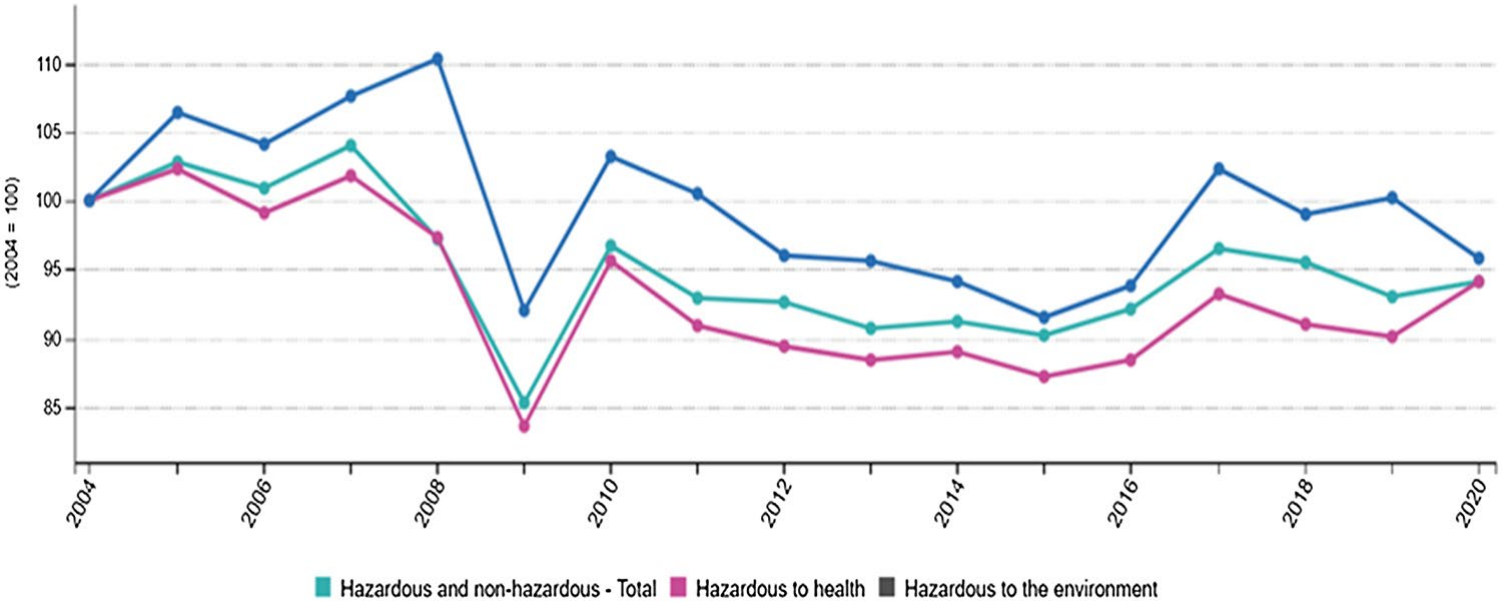
Production of chemicals in the EU (2004–2020; ) https://ec.europa.eu/eurostat/statistics-explained/index.php?title=Chemicals_production_and_consumption_statistics&oldid=548892 source EUROSTAT 2021

During the last 70 years, anthropogenic chemical production has exponentially increased to the point that reasonable estimations indicate a rate of 10 million per year. Global chemical production is thought to grow at a rate of 3% per year, rapidly outpacing the global population growth rate, estimated at 0.77% per year. On this trajectory, chemical production will double by 2024, indexed to 2000 (Wilson and Schwarzman 2009). This fact raises a constant threat for the global population since regulatory agencies will fail to efficiently monitor the rate of introducing such environmental chemicals (ECs) and, more importantly, their potential hazards at exposure doses. In addition, the amount and diversity of pesticides, pharmaceuticals, and other industrial chemicals that humans release into the environment (air, water, soil, and dust) or use for food production make it even more challenging to estimate the actual burden on human health and environmental safety. As shown by the Eurostat report for Chemicals production and consumption statistics, the total production of industrial chemicals in the European Union (EU) increased by 4% from 2004 to 2007, reaching a peak of 314 million tonnes in 2007 (Fig. 1).

During the financial and economic crisis of 2008, production fell and reached a minimum in 2009. The rebound in activity in 2010 was almost as big as the decrease reported in 2009. In 2011, the production of chemicals in the EU decreased again and then increased slightly during 2011–2015, though it was still below the pre-crisis peak in 2007. In 2017, for the first time since 2010, there was a noticeable increase of more than 10 million tonnes. The production of industrial chemicals was concentrated mainly in Western Europe. Notably, the production of chemicals hazardous to health followed a similar trend to that observed for dangerous chemicals to the environment. Production peaked in 2005 and another relative rise in 2007, after which there was a significant decline in production (coinciding with the financial and economic crisis), followed by a strong rebound in 2010. In 2011, the production of chemicals hazardous to health decreased again and continued to decline to a low in 2015. This was followed by a new increase peaking in 2017 (Blum et al. 2017; Eurostat 2019).

The extensive introduction and use of numerous chemicals run parallel to the increased rate (or even emergence) of numerous diseases, particularly chronic diseases. To some extent, this can be attributed to modern lifestyle and environmental hazards. According to the World Health Organisation (WHO), chemicals are responsible for almost 41 million deaths annually, meaning approximately 71% of all deaths registered globally. Cardiovascular disorders are among the top causes of death among chronic diseases, followed by neoplasms, respiratory diseases, and diabetes. Indeed, these four medical conditions represent 80% of all premature deaths by chronic diseases.

The etiology of chronic diseases is a matter of intense research since they represent a complex entity that encompasses several risk factors influenced by various drivers or determinants acting at different levels. Three levels of action have been suggested to play a role in this process; these are proximal (‘cause of the risk factor’), medial (‘cause of the cause’), and distal (‘cause of the cause’) levels (Egger and Dixon 2014). It is noteworthy that due to the research on chronic diseases, the term “para-inflammation” was coined as a new inflammatory state. This describes a state of low-grade, chronic, and systemic inflammation initially observed in obesity and then associated with other chronic pathologies (Hotamisligil et al. 1993; Medzhitov 2008).

It is suggested that exposure to anthropogenic pollutants and their by-products, along with genetic predisposition to chronic diseases and/or epigenetic changes, can increase the susceptibility to chronic diseases. These observations indicate the necessity of using improved hazard-evaluation models, such as the real-life risk simulation (RLRS) scenario (Tsatsakis et al. 2017) to set appropriate regulatory levels for combined chemical exposures. This endeavor entails designing experiments with a combination of chemicals instead of the current testing paradigm of “one chemical-one critical effect” (Docea et al. 2016; Tsatsakis et al. 2016, 2017, 2018, 2019a; Tsatsakis and Lash 2017; Hernández and Tsatsakis 2017).

In an earlier study, we showed that chronic exposure to a chemical mixture closely resembling everyday life exposure, even in very low doses, such as 0.0025 × No-observed-adverse-effect level (NOAEL), can induce cellular alterations in rat organs regardless of sex and some indications of genotoxic and cytotoxic effects (Tsatsakis et al. 2019a). The present study sought to examine whether long-term exposure (18 months) to very low doses of the same mixture of 13 chemicals (below NOAEL levels) induce effects on body weight, organ weight, and histopathological parameters in rats as an animal model. The mixture consisted of carbaryl, dimethoate, glyphosate, methomyl, methyl parathion, and triadimefon, pesticides found as residues in food samples according to the 2013 EU report on pesticide residues in food (Authority EFS 2015) and sodium benzoate, calcium disodium diamine tetraacetate (EDTA), ethylparaben, butylparaben, bisphenol A, aspartame and acacia gum, food and lifestyle additives found in everyday used products (Nishihama et al. 2016; Mantovani 2016; Tzatzarakis et al. 2017; Additives EPoF, Food NSat 2013; Additives EPoF 2017). Notably, the chemicals mentioned above were chosen as standard components of a normal modern lifestyle having as source food, water, and everyday lifestyle products.

## Materials and methods

### Animal study

Twenty male and 20 female 8-week-old Sprague Dawley (CD-SD) rats obtained from the University of Medicine and Pharmacy of Craiova Animal Husbandry were divided into four groups (five males and five females). Animals were acclimatized to laboratory conditions for 2 weeks before the beginning of the experiment (19–23 °C room temperature, 35–55% humidity, and 12 h light/dark cycle). Rats were exposed for 18 months to a mixture of 13 chemicals in four dose ranges as follows: 0×NOAEL (mg/kg BW/day), 0.0025×NOAEL (mg/kg BW/day), 0.01×NOAEL (mg/kg BW/day) and 0.05xNOAEL (mg/kg BW/day) as detailed in previous publications (Tsatsakis et al. 2017, 2019b; Docea et al. 2018, 2019). The mixture contained methomyl, triadimefon, dimethoate, glyphosate, carbaryl, methyl parathion, aspartame, sodium benzoate, EDTA, ethylparaben, butylparaben, bisphenol A, and acacia gum. The corresponding NOAEL values used are reported in detail by Tsatsakis et al. (Tsatsakis et al. 2019a, b). The Ethical Committee of the University of Medicine and Pharmacy of Craiova approved the animal experiment, and all the used procedures were according to the European directive for animal experiments EU Directive 2010/63/EU (European Union 2010).

After 18 months of exposure, animals were anesthetized with a combination of 9.1 mg/kg BW ketamine (Alfasan Int., Woerden, The Netherlands) and 9.1 mg/kg BW xylazine (Alfasan Int., Woerden, The Netherlands) and sacrificed via abdominal aorta exsanguination. The liver, heart, kidneys, spleen, brain, lungs, and testicles in males and uterus in females were collected, rinsed in saline solution and weighed to evaluate changes in relative organ weight (expressed as g/100 g BW).

### Histopathological analysis

Half of the collected organs were fixed in 4% paraformaldehyde for 24 h. They were then dehydrated in a series of ethanol solutions of increasing concentration for 1 h (70% ethanol), 1 h (90% ethanol), and 5 h (100% ethanol). Organs were then fixed in xylene for two hours before being embedded in paraffin. Paraffin blocks were cut into 25 m slices with a microtome and stained with hematoxylin/eosin based on standard protocol (Iordache et al. 2020). A grading system was applied to all tissue specimens to systematically quantify possible histopathological changes. According to this system, Grade 1 describes normal tissue architecture; Grade 2a describes a relative decrease of cellularity ≤ 30%; Grade 2b illustrates the presence of 2 types of parenchyma alterations; Grade 2c describes the presence of both 2a and 2b alterations; Grade 3a describes a relative reduction of cellularity > 30% but ≤ 60%; Grade 3b illustrates s the presence of > 2 types of parenchyma alterations; Grade 3c describes the presence of both 3a and 3b alterations; Grade 4a presents a relative decrease of cellularity > 60% with one type of parenchyma alteration; Grade 4b describes a relative reduction of cellularity > 60% with two types of parenchyma alterations; Grade 4b describes a relative decrease of cellularity > 60% with more than two types of parenchyma alterations and Grade 5 represents the cancerous transformation of the cellular population.

### Statistical analysis

Descriptive statistics of organs’ weight were expressed using means as measures of central tendency and standard deviations or quartiles as measures of dispersion.

The Kolmogorov–Smirnov test was used to test whether relative organ weight (per 100 gr of BW) fitted the normal distribution. Putative changes in organ weights per 100 gr BW were assessed using two-way ANOVA, followed by Dunnet’s and Bonferroni’s ad hoc tests. Multivariate ANOVA was used to determine sex-specific organs (testicles), and one-way ANOVA for the uterus. Additionally, the Kruskal–Wallis was applied to assess potential differences in organ weight/100 g BW between different treatment levels.

IBM SPSS Statistics (version 26.0) was used for data analysis, and a *p* = 0.05 was set as the significance level.

## Results

### Bodyweight and organ weight

Table 1 presents a statistically significant difference in body weight (*p* < 0.001) between male and female rats for the total rat population and the dose groups, except for the control and HD groups. Male rats showed greater weight than females. Further comparison between dose groups regardless of sex (males and females) revealed no significant difference (*p* = 0.599). The control group had the highest weight for females, while the HD group showed the highest weight among the treated groups. For males, the LD group showed the greatest weight.

**Table 1:** Rats’ weight per group and sex

Group	Gender						*P*
	Female		Male		Total		
	Mean	SD	Mean	SD	Mean	SD	
Control	452.2	99.5	546.8	92.5	499.5	103.4	Illustrates
LD	389.0	67.6	677.6	128.9	533.3	180.4	0.002
MD	426.4	60.6	515.8	59.7	471.1	73.7	0.047
HD	432.4	39.0	596.4	196.8	514.4	159.2	0.105
Total	425.0	68.5	584.2	134.7	504.6	132.7	< 0.001
	*F*(3, 32) = 0.633, *p* = 0.599, *F*(1, 32) = 23.259, *p* < 0.001						

Table 2 shows the descriptive statistics of the relative organ weight of animals treated with different doses of the mixture. The interaction of dose and sex on organ weight was statistically significantly reduced for the heart (*p* = 0.029) and lung weight (*p* = 0.01) (Table 2). For females, the liver showed higher weight in the control and MD groups; the right kidney had a greater weight in the control and LD groups; the left kidney had the greatest weight in the LD group; the heart in the LD group; the spleen had equal weight in the control and LD groups; the brain had the greatest weight in the LD group, as well as the lungs and pancreas. For males, the liver showed a greater weight in the LD group; both kidneys showed higher weight in the MD group; the heart was found heavier in the MD group; the spleen was found to be heavier in the control group while from the exposure groups in the HD groups; the brain was found to be heavier in the MD group, the lungs were found to be heavier in the HD group and the pancreas was found to be heavier in the HD group. Further statistical analysis in which comparison between exposure groups when both sex and dose were taken into account [F(dose/sex)] revealed that the liver is significantly lighter in the female HD group and significantly heavier in the LD group; the right and the left kidneys did not show significant differences of their weight among females while for males they were found to be significantly heavier in the MD group; the heart was found to be heavier among the LD group while no significant difference was found among males; the spleen was found to be lighter among males of the LD group while no significant difference was found among females; the brain was found to be heavier among females of the LD group while no significant difference was found among males; the lungs were found to weight substantially more among the females of the LD and HD groups while for the male group lungs were found to be heavier among the MD and HD groups; the pancreas was found to weight more among the females of the HD group and markedly less among the females of the MD group while the only significant difference among males was found at the HD group were it weighted more.

**Table 2:** Descriptive statistics of relative weight at different doses of chemicals mixture

Organ relative weight (g/100 g bw)	Dose	Sex				Effects		
		Female		Male		*F* (dose)	*F* (sex)	F (D × sex)
		Mean	SD	Mean	SD	*df* (3.32)	*df* (1.32)	
Liver	Control	2.59	0.39	2.25	0.16	*F* = 2.97	*F* = 1.2	*F* = 0.86
	LD	2.52	0.16	2.49	0.34	*p* = 0.046	*p* = 0.281	*p* = 0.47
	MD	2.53	0.18	2.47				
	HD	2.16	0.27	2.19	0.28			
Kidney Right	Control	0.38	0.20	0.37	0.04	*F* = 0.54	*F* = 1.19	*F* = 0.71
	LD	0.35	0.09	0.35	0.05	*p* = 0.657	*p* = 0.283	*p* = 0.551
	MD	0.33	0.04	0.43	0.04			
	HD	0.32	0.02	0.35	0.08			
Kidney Left	Control	0.32	0.12	0.38	0.03	*F* = 0.73	*F* = 5.34	*F* = 0.46
	LD	0.35	0.09	0.36	0.04	*p* = 0.542	*p* = 0.027	*p* = 0.709
	MD	0.33	0.04	0.41	0.06			
	HD	0.30	0.02	0.35	0.09			
Heart	Control	0.25	0.04	0.24	0.03	*F* = 0.59	*F* = 5.02	*F* = 3.4
	LD	0.31	0.05	0.22	0.02	*p* = 0.628	*p* = 0.032	*p* = 0.029
	MD	0.25	0.03	0.25	0.03			
	HD	0.25	0.02	0.24	0.06			
Spleen	Control	0.21	0.13	0.20	0.08	*F* = 0.7	*F* = 0.45	*F* = 1.04
	LD	0.21	0.03	0.14	0.01	*p* = 0.559	*p* = 0.507	*p* = 0.39
	MD	0.17	0.02	0.17	0.03			
	HD	0.17	0.04	0.19	0.04			
Brain	Control	0.40	0.10	0.33	0.05	*F* = 0.74	*F* = 11.74	*F* = 0.8
	LD	0.48	0.13	0.32	0.07	*p* = 0.537	*p* = 0.002	*p* = 0.503
	MD	0.45	0.06	0.39	0.06			
	HD	0.42	0.02	0.35	0.11			
Lung	Control	0.91	0.42	1.09	0.57	*F* = 2.38	*F* = 0.1	*F* = 4.41
	LD	2.10	0.78	0.79	0.35	*p* = 0.088	*p* = 0.752	*p* = 0.01
	MD	1.06	0.33	1.43	0.87			
	HD	1.33	0.53	2.40	1.47			
Pancreas	Control	0.35	0.10	0.30	0.05	*F* = 4.1	*F* = 1.82	*F* = 2.33
	LD	0.37	0.14	0.27	0.08	*p* = 0.014	*p* = 0.187	*p* = 0.093
	MD	0.24	0.03	0.33	0.09			
	HD	0.50	0.11	0.37	0.15			

Table 3 presents the descriptive statistics of reproductive organs per dose of a chemical mixture. As shown, there is no statistically significant difference for any of the organs between the different dose groups.

**Table 3:** Relative weight of reproductive organs per dose of chemicals mixture

Organ relative weight (g/100 g bw)	Dose	Mean	SD	*F* (dose)	*p*
Testicle right	Control	0.283	0.052	*F* = 0.027	0.994
	LD	0.276	0.011		
	MD	0.277	0.037		
	HD	0.282	0.069		
Testicle left	Control	0.273	0.045	*F* = 0.588	0.63
	LD	0.242	0.024		
	MD	0.273	0.038		
	HD	0.283	0.081		
Uterus	Control	0.251	0.097	F = 0.575	0.64
	LD	0.288	0.197		
	MD	0.203	0.070		
	HD	0.210	0.041		

### Histopathology findings

Table 4 presents the histopathological findings in the four groups of exposed animals while Table 5 presents the histopathological assessment of the organ tissues expressed using the grading system described in the materials and methods section.

**Table 4:** Histopathological findings in the four groups of animals

Organ	Histology control	Histology 0.0025 noael	Histology 0.01 noael	Histology 0.05 noael
	M&F	M&F	M&F	M&F
Testis	Normal architecture, cellularity of Leydig, Sertoli, and germ cells	Mild architectural distortion, hemorrhagic infiltrations, and mild to a moderate decrease in cell number of all cell types	Moderate architectural distortion, hemorrhagic infiltrations, and mild to moderate reduction in the number of all cell types	Testis with extended architectural distortion, hemorrhagic infiltrations,and a significant reduction in the number of all cell lines
Liver	The normal architecture of sinusoidal spaces, bile canaliculi, normal cellularity,and no sign of inflammation	Mild distortion of the architecture with accompanying focal parenchymal and portal canal lymphocytic infiltration	Moderate distortion of the architecture with accompanying focal parenchymal and portal canal lymphocytic infiltration and multinucleosis of hepatic cells	Severe distortion of the architecture with accompanying parenchymal and portal canal lymphocytic infiltration with mild disruption of the terminal plate and anisonucleosis and multinucleosis of hepatic cells
Heart	Normal architecture and cellularity with no sign of inflammation	Mild distortion of the muscle architecture and inflammation	Moderate distortion of the muscle architecture and inflammation	Severe distortion of the muscle architecture and inflammation
Lung	Normal bronchial and bronchoalveolar architecture with the limited inflammatory response (presence of lymphocytes) in the peribronchial tissue	Mild distortion of the architecture and increase of the peribronchial inflammation (elements indicative of mild bronchopneumonia and bronchoalveolitis)	Moderate distortion of the architecture, intense presence of alveolar macrophages with phagocytic activity, and intense peribronchial inflammation (elements indicative of moderate bronchopneumonia and bronchoalveolitis)	Severe distortion of the architecture, the highly intense presence of alveolar macrophages with phagocytic activity, and intense peribronchial inflammation (elements indicative of moderate bronchopneumonia and bronchoalveolitis)
Muscle	Normal architecture of muscle fibers with no inflammatory elements	Mild derangement of muscle fibers	Moderate derangement of muscle fibers	Severe derangement of muscle fibers
Brain	Normal architecture, glial cellularity with no sign of inflammation	Mild distortion of the architecture and mild decrease of glial cells with inflammation	Moderate distortion of the architecture and moderate reduction of glial cells with parallel inflammation	Severe distortion of the architecture and severe reduction of glial cells with parallel inflammation
Kidney	Normal architecture of the cortical, medullar portion, pelvis, and calyxes with no indication of inflammation	Mild inflammatory cell infiltration and distortion of glomerular and renal tubule architecture	Mild inflammatory cell infiltrate and mild distortion of glomerular and renal tubule architecture and presence of tubular casts	Mild to moderate inflammatory cell infiltrate and mild distortion of glomerular and renal tubule architecture with single-cell tubular necrosis and presence of tubular casts
Stomach	Normal architecture with no distortion of the mucosal integrity and no signs of inflammation	Mild distortion of mucosal architecture and few mucosal foci of necrosis with inflammatory cell infiltrate	Moderate distortion of mucosal architecture and necrotic lesions of mucosa with inflammatory cell infiltrate	Severe distortion of mucosal architecture and necrotic lesions of mucosa with inflammatory cell infiltrate
Pancreas	The normal architecture of lobules and islets of Langerhans with no inflammation	Mild distortion of architecture and mild lymphocytic inflammatory response	Moderate distortion of architecture and moderate lymphocytic inflammatory response	Severe distortion of architecture and moderate lymphocytic inflammatory response
Spleen	Normal architecture with dense cellularity of the red and the white pulp	Mild distortion of the architecture, mild decrease of the white pulp volume and mild increase of the red pulp volume	Moderate distortion of the architecture, moderate reduction of the white pulp volume, and moderate increase of the red pulp volume	Severe distortion of the architecture, a prominent decrease of the white pulp volume, and a pronounced increase in the red pulp volume

**Table 5:** Histopathological assessment of the organ tissues using our grading system

	Control	0.0025 × noael	Number of affected subjects	0.01 × NOAEL	Number of affected subjects	0.05 × NOAEL	Number of affected subjects
Testis	I (5/5)	IIc	3/5	IIIc	4/5	IVc	5/5
Liver	I (10/10)	IIc	9/10	IIIc	9/10	IVc	10/10
Heart	I (10/10)	IIa	8/10	IIIa	7/10	IVa	9/10
Lung	I (10/10)	IIa	9/10	IIIc	10/10	IVc	10/10
Muscle	I (10/10)	IIa	7/10	IIIa	8/10	IVa	6/10
Brain	I (10/10)	IIa	8/10	IIIc	7/10	IVb	8/10
Kidney	I (10/10)	IIc	10/10	IIIc	10/10	IVc	10/10
Stomach	I (10/10)	IIc	8/10	IIIc	9/10	IVb	8/10
Pancreas	I (10/10)	IIb	7/10	IIIc	7/10	IVb	8/10
Spleen	I (10/10)	IIc	7/10	IIIc	9/10	IVc	7/10

As both sexes exhibited the same extent of parenchyma and stroma deterioration, organ findings are presented for both sexes as a single category. A dose-dependent effect is revealed as well as a mild organ-specific effect. Overall, the testes, liver, lungs, kidneys and spleen presented the more severe deterioration, followed by the stomach, pancreas and brain presenting an intermediate overall distortion and finally the heart and striated muscle, which were the less affected.

Histopathological assessment of the organ tissues revealed organ-specific pathological findings. Thus, comparative histopathological evaluation of the testes between the control and dose groups revealed a dose-dependent deleterious effect of the mixture regarding the degree of tissue disfigurement. The testicular toxicity observed is nonspecific, affecting all germ cell types. Inflammatory infiltration was not observed, which is well correlated with the absence of disruption of seminiferous tubules. The histopathological evaluation of the liver tissue showed a change in liver cells' tinctorial qualities, observed as more eosinophilic granular cytoplasm in the medium and high doses. Also, the 0.01 × NOAEL and 0.05 × NOAEL dose groups exhibited karyomegaly, multinucleosis, and anisonucleosis of the hepatocytes. Finally, lymphocytic infiltration was evident focally in liver parenchyma and portal tracts with focal disruption of the terminal plate in the high dose group. The above morphologic alterations exhibited a dose-dependent manner of presentation.

Heart tissue evaluation revealed that both sexes were equally affected by the chemical mixture with a steady decline of the organs’ normal histology and signs of atrophy of heart muscle tissue accompanied by infiltration of lymphocytes in a dose-dependent manner.

In lung tissue, a dose-dependent tissue histology distortion, inflammatory cells infiltration and enhanced phagocytic activity by alveolar macrophages were observed in male and female rats. Likewise, an enhanced phagocytic activity by alveolar macrophages was detected in 0.0025 × NOAEL, 0.01 × NOAEL, and 0.05 × NOAEL.

Histopathological examination of striated muscle showed a dose-dependent effect on the tissue structure with a derangement of muscle fibers without any signs of inflammation. On the other hand, exposure to the chemical mixture induced structural deformities, morphological changes, and inflammation of the brain tissue. A dose-dependent decrease in glial and astrocyte cells’ number was evident, resulting in net atrophy of the cortical and subcortical parenchyma. Also, a mild inflammatory cell infiltration was observed in all treated groups of rats.

**Fig. 2:**
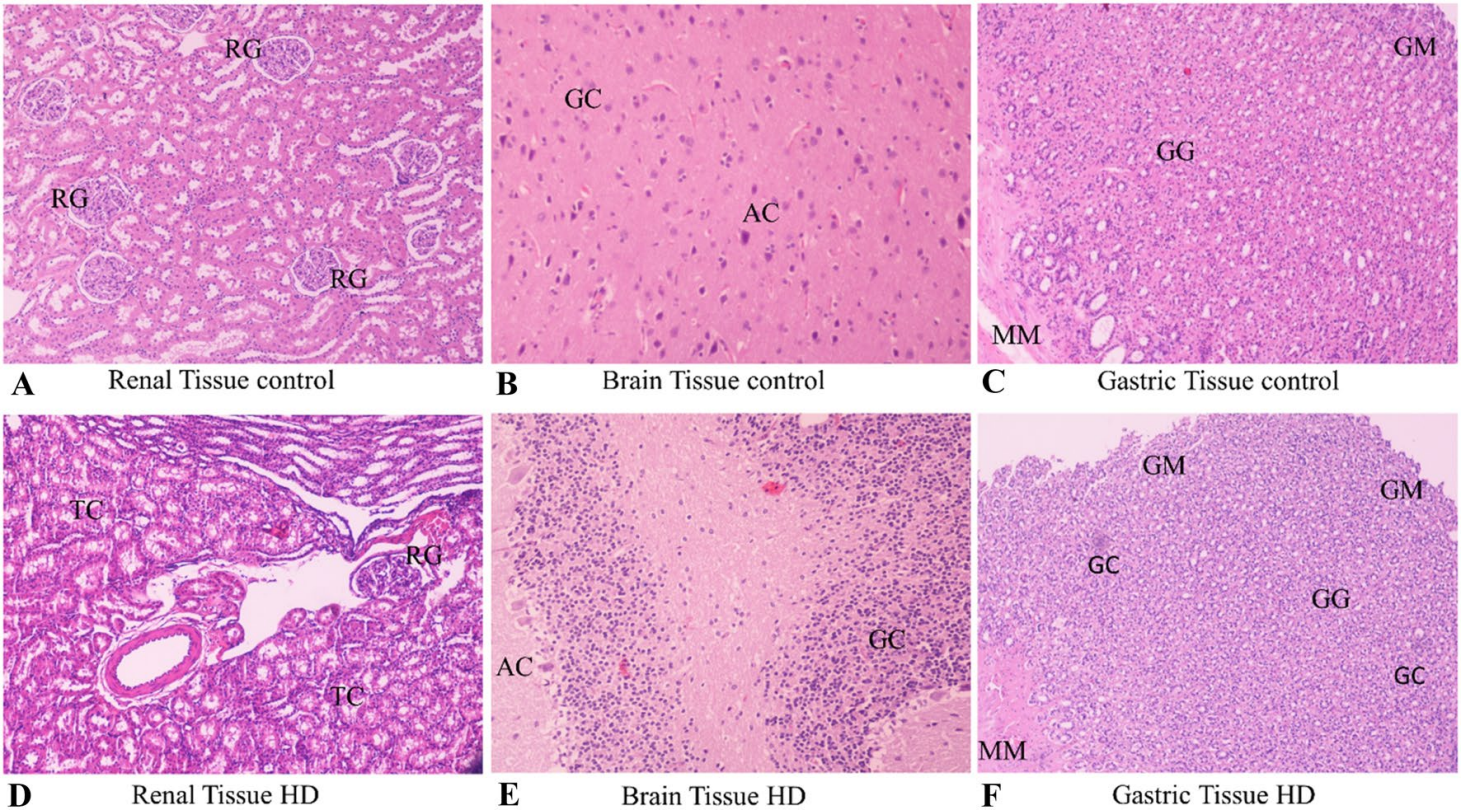
Slides of renal, brain and gastric tissue stained with eosin hematoxylin of control and high dose subjects. Magnification × 200. **A** Renal tissue control × 200. *RG* Renal glomerulus. Normal shape and number of renal glomeruli with normal cellularity level. **B** Brain tissue control × 200, *AC* astrocytes, *GC* glial cells. Normal number of glial cells and astrocytes. **C** Gastric tissue control × 200 *GM* Gastric mucosa, *GG* Gastric glands, *MM* Muscularis mucosae with normal architecture with no distortion of the mucosal integrity and no signs of inflammation. **D** Renal tissue high dose (HD) × 200. *TC* Tubular cells. Severe numerical reduction in cellularity with intense growth in tubular cell aggregates forming renal tubules molds. **E** Brain tissue high dose (HD) × 200, *AC* astrocytes, *GC* glial cells. Severe numerical reduction of glial and astrocyte cells. **F** Gastric tissue high dose (HD) × 200 *GM* Gastric mucosa, *GG* Gastric glands, *GC*  granulocytes, *MM* Muscularis mucosae with severe distortion of mucosal architecture and necrotic lesions of mucosal foci along with mucosal degeneration

A dose-dependent histological damage of glomeruli and renal tubules was observed in the kidneys for the three treatment groups accompanied by a mild lymphocytic infiltration (Fig. 2).

Evaluation of the stomach tissue morphology revealed degenerative changes of epithelial cells and a few necrotic lesions accompanied by inflammation. These effects were determined to be dose-dependent. No differences were found to be sex-related (Fig. 2).

Examination of pancreatic tissue unveiled a dose-dependent disfigurement of tissue structure and a lymphocytic inflammatory response with no differences between male and female animals.

Microscopic examination of the spleen showed an alteration of tissue histology, with a decrease in the white pulp volume and an increase in the red pulp volume. The above changes were more pronounced in the animal groups treated with the medium and high doses.

## Discussion

Owing to the tremendous increase in chemical use in everyday life, modern societies face an unparalleled threat of numerous public health issues. In order to deal with this increasing threat, strict legislation for chemical use has been enforced, and numerous chemicals have been restricted or even banned from use. However, the public is yet to be effectively protected. It is indicative that the incidence of neurodegenerative disorders, cardiovascular diseases, obesity, and metabolic syndrome, infertility (primarily in men), and inflammatory conditions of any nature have dramatically increased (Inhorn and Patrizio 2015). Interestingly, during the last decade, various studies proved the toxic hazard of multiple chemicals for public health even at levels that were, until then, considered to be safe for human use (Yang et al. 2017). This means that the general population is exposed to a delayed wash-out from the harmful chemicals used in the past and those currently used. Notably, it should be considered that contemporary people are not exposed to single chemicals but chemical mixtures. Even though these mixtures most often consist of chemicals in levels well-below safety regulations, chronic exposure to them raises specific safety concerns. Indeed, it is suggested that this chronic exposure to low concentrations of various chemicals allows the incriminated substances to distort the protective mechanisms and homeostasis and, thus, lead to chronic diseases.

A drawback of most research protocols addressing the issue of exposure to low doses of chemicals is using only a single or a small mix of chemicals for a relatively short period of time. Due to the limitations of the study protocols, no firm conclusions can be drawn regarding the actual effect of this type of exposure on human health. Thus, the study's driving force was to obtain data mimicking chronic exposure to a larger mix of substances that correlates better with real-life conditions. It has been well-documented that long-term exposure to low doses of chemical mixtures can seriously threaten an organism’s health. In addition, a growing body of evidence manifests the potential risk of various chemicals acting as cellular modifiers and determinants of numerous contemporary diseases, especially autoimmune ones. Therefore, real-life risk simulation experimental models represent an efficient tool for validating a compound’s actual effects better resembling real-life conditions (Tsatsakis et al. 2017). Our study focuses on a 13 compound-mix [six pesticides (carbaryl, dimethoate, glyphosate, methomyl, methyl parathion, and triadimefon) and seven food and lifestyle additives (sodium benzoate, calcium disodium ethylene diamine tetra acetate (EDTA), ethylparaben, butylparaben, bisphenol A, aspartame and acacia gum] to which individuals living in contemporary societies are often exposed.

Carbaryl is an insecticide used on various crops such as corn, soybean, cotton, fruit, nut, and vegetable crops, as well as in-home yards and gardens. Acute (short-term) and chronic (long-term) occupational exposure to carbaryl has been observed to cause cholinesterase inhibition, and reduced levels of this enzyme in the blood is considered a proxy of neurological effects. These effects appear to be reversible upon discontinuation of exposure. Headaches, memory loss, muscle weakness and cramps, and anorexia are caused by prolonged low-level exposure to carbaryl resulting from cholinesterase inhibition. The United States Environmental Protection Agency (EPA) has classified carbaryl as a Group D, not classifiable as to human carcinogenicity (United State Environmental Protection Agency (US EPA) 2000).

Dimethoate is a systemic and contact organophosphorus insecticide registered for use in the US in 1962 and used on several field-grown agricultural crops (e.g., leaf greens, citrus, and melons), tree crops, and ornamentals. Residential and non-agricultural uses were canceled in 2000. In the EU, however, the approval of the active substance dimethoate was not renewed by Commission Implementing Regulation (EU) 2019/1090. Use on alfalfa, wheat, cotton, and corn crops account for more than 60% of the total dimethoate use in the US (US EPA 2006). Dimethoate is absorbed from the gastrointestinal tract, the lungs, and through the skin. General population exposure to dimethoate can occur by eating foods treated with dimethoate or drinking water. Among professional pesticide applicators and formulators, dimethoate exposure can occur by skin contact or inhaling aerosols or dust (CDC 2013).

Glyphosate is a widely used herbicide that controls broadleaf weeds and grasses. It has been registered as a pesticide in the US since 1974. Glyphosate targets a broad range of weeds and is important in producing fruits, vegetables, nuts, and glyphosate-resistant field crops such as corn and soybean. It is effective at managing invasive and noxious weeds. In addition, glyphosate breaks down in the environment, can be used for no-till and low-till farming, which can reduce soil erosion, and is useful for integrated pest management. According to EPA, when glyphosate products are used according to label directions do not result in risks to children or adults. Moreover, the EPA states that there is no indication that children are more sensitive to glyphosate from in-utero or post-natal exposure, nor that glyphosate causes human cancer (US EPA 2022). Methomyl is a carbamate insecticide that controls foliage and soil-borne insect pests on various food and feed crops. Methomyl is used mostly in agriculture and is currently registered for use on field vegetables like lettuce, orchard crops like oranges, and on turf (sod farms only). The only non-agriculture use of methomyl is as a fly bait product, which is not restricted to use. Methomyl is extremely toxic if ingested and moderately toxic if inhaled. It is a cholinesterase inhibitor in humans at high enough doses; that is, it can overstimulate the nervous system resulting in nausea, dizziness, confusion, and at very high exposures (e.g., accidents or major spills), respiratory paralysis, and death. To minimize health impacts, EPA classified methomyl products used in agricultural settings as “restricted use,” meaning they can be used only by or under the direct supervision of specially trained and certified applicators (US EPA 2021).

Methyl parathion is a pesticide used to kill insects on crops. Usually, it is sprayed on the crops. Methyl parathion is no longer used on food crops commonly consumed by children. The maximum amount of methyl parathion that can be present as a residue on specific crops is regulated. Exposure to very high levels of methyl parathion for a short period in air or water may cause death, loss of consciousness, dizziness, confusion, headaches, difficulty breathing, chest tightness, wheezing, vomiting, diarrhea, cramps, tremors, blurred vision, and sweating. Some people exposed to substances similar to methyl parathion have experienced changes in their mental state that lasted several months after exposure to high levels of these substances ended. If people are exposed to levels of methyl parathion below those that affect nerve function, few or no health problems seem to occur. There is no evidence that methyl parathion causes congenital disabilities in humans or impairs fertility. There is also no proof that methyl parathion causes cancer in regularly exposed people, such as farmers and pesticide applicators (ATSDR 2001). Triadimefon is a broad spectrum, systemic fungicide called triazoles. Triadimefon controls various fungal diseases in fruit (pineapple) and non-food use sites such as pine seedlings, Christmas trees, residential (sod farm), and commercial turf, ornamentals, and landscapes. The endpoint of concern for triadimefon is neurotoxicity, observed in rat, mice, and rabbit studies. Moreover, the Cancer Assessment Review Committee (CARC) assigned triadimefon a classification of Category C “possible human carcinogen” (US EPA 2006). In 2016, the European food safety authority (EFSA) Panel on Food Additives and Nutrient Sources added to Food (ANS) was asked to deliver a scientific opinion re-evaluating benzoic acid (E 210), sodium benzoate (E 211), potassium benzoate (E 212) and calcium benzoate (E 213) when used as food additives. Benzoic acid and its sodium and potassium salts are rapidly absorbed after oral administration. Absorption, distribution, metabolism, and excretion of calcium benzoate will be similar to sodium or potassium salt; therefore, read-across between the salts was possible. The results of short-term and subchronic studies on benzoic acid and its salts indicate their toxicity is low. Using benzoic acid and its sodium and potassium salts as food additives do not raise concerns concerning genotoxicity. Also, this conclusion is applicable to calcium benzoate. Moreover, the available data did not indicate any carcinogenic potential (Additives EPoF, Sources N 2016).

Parabens are man-made chemicals often used in small amounts as preservatives in cosmetics, pharmaceuticals, foods, and beverages. Common parabens are methylparaben, ethylparaben, propylparaben, and butylparaben. Often more than one paraben is used in a single product. People can be exposed to parabens through touching, swallowing, or eating products that contain parabens. Many products, such as makeup, moisturizers, hair-care products, and shaving creams, contain parabens. Parabens in these products are absorbed through the skin. Parabens also can enter the body when pharmaceuticals, foods, and drinks containing parabens are swallowed or eaten. In 2006, the industry-led Cosmetic Ingredient Review (CIR), in partnership with the US Food and Drug Administration (FDA), determined that there was no need to change CIR’s original conclusion from 1984 that parabens are safe for use in cosmetics. The FDA allows single or multiple parabens to be added to food or food packaging as antimicrobials to prevent food spoilage (CDC 2017).

Bisphenol A (BPA) is a structural component in polycarbonate beverage bottles. It is also a component in the metal can coatings, which protect the food from directly contacting metal surfaces. BPA has been used in food packaging since the 1960s. BPA poses no health risk to consumers because current exposure to the chemical is too low to cause harm. EFSA’s scientific opinion shows that the level of BPA that consumers of all ages are exposed to is well below the estimated level of safe exposure—known as the tolerable daily intake (TDI). EFSA finds there is no health concern as the highest estimates for dietary and aggregate exposure to BPA are 3–5 times lower than the TDI, depending on the age group. For all population groups, dietary exposure on its own is more than fivefold below the TDI (EFSA Panel on Food Contact Materials E, Flavourings, Aids P 2015).

Taking all the above information into account, we utilized cytological and pathological examination to determine putative disfigurement of treated animals’ tissues. Organs’ weight in male rats was higher than that of females overall, while the greatest weight was shown by the LD group. Moreover, the organs that demonstrated the most significant weight difference (when both sex and dose were taken into account) were the heart and the lungs of the LD group. Following pathology evaluation, the LD group showed a mild worsening of all parameters, a shared common manifestation being the chronic inflammatory infiltration indicating a mild but generalized inflammation. On the other hand, the MD group exhibited a further worsening of all parameters, including the disfiguration of the histological architecture. The most severely affected organs were the lungs, the liver, the kidneys, and the stomach exhibiting chronic respiratory disease (due to the infiltration of inflammatory cells owing to significant distortion of lung parenchyma with alteration of the bronchoalveolar architecture in addition to the enhanced phagocytic activity by alveolar macrophages that was detected suggesting increased permeability of the alveolocapillary membrane with concomitant dysfunction) mild hepatitis/cholangitis, glomerulonephritis /chronic renal tubular necrosis, and gastric ulcers. However, it should be noted that even though both cytological and pathological testing revealed the deleterious effect of the chemical mixture on the various tissues, the cellular and architectural distortion (when present) could in fact, be identified in greater detail by histopathology unveiling the actual extent of the damage. Therefore, the current study was able to identify an increased organ/tissue distortion in both sexes and especially in high-dose groups, while in our previous study using cytology, only females exhibited such an effect.

Inflammatory infiltration was even more pronounced in the HD group, possibly associated with a generalized response of the immune system to noxious stimuli. In addition to the histopathological distortion, these findings suggest that sustained inflammation might overwhelm the organism’s repairing mechanisms in the long term, thus resulting in permanent damage. In the brain tissue, both cortical and subcortical areas showed severe distortion of their architecture and a decrease in a neural cell population, suggesting a possible predisposition for neurodegenerative disorders at a later life stage.

Heart function was also compromised since its architecture was severely damaged. Moreover, even though a net decrease in body weight would be expected, rats very likely might have suffered from some kind of myositis which, along with heart failure, could predispose them to increased body weight due to lack of exercise. As the renal function was also affected according to the signs of renal tubular injury, liquid retention might also have contributed to the increased body weight. However, since rats did not present any symptoms from their urinary tract before they were sacrificed, this renal injury should not have a major effect on them.

The sexual function should also be compromised since all germ lines were decreased, including Leydig cells, which are responsible for producing testosterone. Thus, a relative testosterone/estrogen imbalance in males, along with the estrogen-mimicking compounds (parabens) that were administered, might contribute to reproductive toxicity. Finally, the increase of the spleen’s red pulp and decrease of white pulp volume can be consistent with a generalized inflammatory response induced by exposure to the chemical mixture. This may have resulted in red pulp hypertrophy (leading to an expected drop in whole WBCs count) and white pulp shrinkage (which is expected to be associated with low lymphocyte count). The control group was found to be within normal limits for all studied parameters both on cytology and pathology examination. The only notable event was the slight peribronchial presence of lymphocytes suggesting subclinical bronchitis. These findings correlate well with our previous findings that evaluated the genotoxic, cytotoxic, and cytopathological effects of the same chemical mixture over the same period of time (Tsatsakis et al. 2019a). However, in our previous study, the extent of organ lesions was not as evident as in this one. This could be due to the method used to assess those lesions (cytopathology hematoxylin and eosin staining lacks the ability to thoroughly examine tissue in depth and is most often affected by red blood cell contamination of the examining field). In 2015, Goodson et al., along with a panel of experts, assessed the carcinogenic potential of low-dose exposures to chemical mixtures. Based on this evaluation, various substances such as bisphenol A, were categorized as being potentially carcinogenic through a multi-pathway mechanism (Goodson et al. 2015). Buñay et al. also assessed the effect of potent environmental endocrine disruptors such as phthalates after chronic low-dose exposure. miRNA/isomiRs deregulation, along with histopathological changes in tested and hormonal alterations, were identified in adult mice (Goodson et al. 2015). Recently, Ghasemnejad-Berenji et al. proved that a five pesticides mixture could induce oxidative stress and impaired memory and learning ability in rats. Taking all this evidence into account, some additional conclusions can be drawn (Ghasemnejad-Berenji et al. 2021).

First, the extensive deterioration in organ architecture observed in the main sites of chemical metabolism of the HD group (such as the liver, kidneys, and lungs) could be considered an early indication of potential carcinogenesis in these organs. However, no cancer formation was observed, which may be due to the relatively short period of exposure or because anti-carcinogenic genes (for example, that of p53 or mTOR) may not have been affected yet (Darbre and Harvey 2014; El-Shennawy et al. 2020). In addition, the study design (doses used, number of animals per dose, duration of the treatment) was not appropriate to assess carcinogenesis. On the other hand, the decreased brain cellularity and deregulation of brain architecture can explain the impairment of mental status that Ghasemnejad-Berenji et al. identified in their study and might be due to the well-known side effects of many pesticides that we included in our mixture. In addition, our previous study identified that this mixture was able to induce genotoxic effects in MD and HD groups, as evidenced by the micronuclei assay.

Though we did not run a genetic analysis to identify potential gene mutations, these findings are consistent with previous studies showing that long-term exposure to low doses of pesticides impairs DNA integrity (Alleva et al. 2018). Finally, in our study, both sexes were equally affected by the harmful effect of the chemical mixtures. This suggests that neither the greater metabolic capabilities of male rats nor the protective effects of the female estrogen hormone system could effectively stand against the detrimental effect of the chemical mixture (Della Torre 2020; Kulkarni 2009; Shimizu and Ito 2007). The latter could be due to the fact that some of the chemicals tested have been identified to act as endocrine disruptors (such as bisphenol A, triadimefon, ethylparaben, and butylparaben) and, therefore could have caused a deregulation of the endocrine system (Jiang et al. 2017; Nowak et al. 2018; Rubin 2011). Lastly, it has to be noted that as evidenced by a recent systematic review on low-dose exposure to chemical mixtures in mammalian in vivo systems, the combination of the chemicals used in this study is unique since no other study has used such a complex but also real life simulating mixture before (Elcombe et al. 2022). Thus, the conclusions drawn from this study are pivotal, and further experiments using an animal model of younger age or even pregnant females would provide further insight.

This study has two main limitations. First, not all organs were harvested due to technical limitations and therefore, no safety conclusions can be drawn regarding the actual effect of this mixture to rats’ organism over 18 months. Second, since our study required the sacrifice of animals for histopathological examination of organs, we have no data on whether the lesions identified are reversible or not. Such an observation could be achieved by acquiring biopsies from certain organs (like skeletal muscles or the liver) during the exposure period and/or if animals were allowed to recover over a certain wash-out period of no exposure. Hopefully, such experiments will be performed in future studies.

A general limitation of the present study is that, despite having used a mixture of 13 chemical substances intended to simulate human exposure in the most reliable way, such mixture was administered to rats at daily constant doses over 18 months. However, humans do not show such uniform exposure and are rather intermittently exposed to variable doses of chemical substances over different periods of time, which cannot be reproduced in long-term studies with animal models. Given the infinite combinations of chemicals and doses that humans can be exposed to throughout a lifetime, the only feasible way to model human exposure is by using physiological-based kinetic (PBK) models based on computational approaches.

## Conclusions

This experimental study shows that exposure to very low doses of a mixture of 13 chemicals induced histopathological and organ weight adverse effects in both sexes. Even though the female genitalia was not evaluated, testicles were affected in a dose-dependent manner since both cellularity and architectural integrity have deteriorated. Exposure to the chemical mixture also affected the liver, stomach, lungs, brain, kidneys, heart, spleen, pancreas, and muscles a dose-dependent manner in both sexes. Overall, this study supports the hypothesis that long-term exposure to chemicals from everyday life, even at very low doses considered as safe, can lead to adverse effects. The current approach for the determination of safety levels of chemicals should be updated to account the real-life exposure scenarios and to shift from the single-chemical approach to real-life risk simulation approach.

## Data Availability

The dataset presented in this study is available from the corresponding author upon reasonable request.
